# Prebiotic, Antioxidant, and Immunomodulatory Properties of Acidic Exopolysaccharide From Marine *Rhodotorula* RY1801

**DOI:** 10.3389/fnut.2021.710668

**Published:** 2021-08-23

**Authors:** Zheng Wang, Yanchen Zhao, Yan Jiang, Weihua Chu

**Affiliations:** ^1^State Key Laboratory of Natural Medicines, School of Life Science and Technology, China Pharmaceutical University, Nanjing, China; ^2^Animal, Plant and Food Inspection Center of Nanjing Customs, Nanjing, China

**Keywords:** acidic exopolysaccharide, prebiotic, antioxidant, immunomodulatory, *Rhodotorula*

## Abstract

In this study, an extracellular acidic polysaccharide (EAPS) from marine *Rhodotorula* sp. RY1801 was extracted, and its biological properties were investigated. EAPS is mainly composed of monosaccharides, including mannose, rhamnose, glucose, galactose, and fucose, had an average molecular weight of 5.902 × 10^7^ Da. The results indicated that EAPS can promote the growth of *Lactobacillus acidophilus* and *L. acidophilus plantarum*. EAPS is capable of scavenging both superoxide anion and hydroxyl radicals *in vitro*. The highest scavenging rate of superoxide anion and hydroxyl radicals is 29 and 84%, respectively. Using *in vivo* model, we found that the EAPS can expand the lifespan and increase the disease resistance of *Caenorhabditis elegans* against *Klebsiella pneumoniae* infection *via* the DAF-2/DAF-16 pathway. These results suggested that EAPS from marine *Rhodotorula* sp. RY1801 could promote the growth of beneficial bacteria and can be used as an antioxidant and immunomodulator, which had considerable potential in the food and health industry.

## Introduction

A polysaccharide is formed by linking 10 or more monosaccharides to each other through glycosidic bonds. Since the 1950s, the study of polysaccharides has aroused the interest of researchers and has gradually become a hotspot (1). After an in-depth study of various polysaccharides, researchers found that natural polysaccharides, like *Flammulina velutipes* polysaccharide, *Dendrobium huoshanense* polysaccharide, *Sargassum horneri* polysaccharide, *Moringa oleifera* polysaccharide, *Ganoderma lucidum* polysaccharide, and *Astragalus* polysaccharide, have good biological activities, such as antitumor, immunomodulation, antiviral, antibacterial, antioxidant, hypoglycemic, hypolipidemic, and some other functions (2). More than 300 natural polysaccharides have been identified, which come from different plants, animals, and microorganisms (3, 4). Microbial polysaccharides, in general, can be divided into three categories, namely, extracellular polysaccharides (EPSs), cell wall polysaccharides, and intercellular polysaccharides (5). In addition to bacterial capsule polysaccharides, many other microbial polysaccharides are of interest to researchers, some of which have been maturely developed for use in the food, agriculture, and healthcare industries. For example, in the food industry, microbial polysaccharides are often used as stabilizers, thickeners, gelling agents, and processing excipients to improve the quality of food (6, 7), alginate and xanthan gum can be obtained from algae and microorganisms, which are used in dairy products, beverages, sauces, bread, frozen foods, and meat and other food processing to improve the texture and taste of food (8). Microbial polysaccharides are not only prominent in food and agriculture but also play a very important role in the healthcare industry.

Various fungal species including *Saccharomyces cerevisiae, Candida albicans*, and *G. lucidum* can produce polysaccharides (9, 10). Yeast EPS is a type of microbial polysaccharide with a variety of potential activities, such as antiviral, antithrombotic, and antioxidant, in addition to promising applications in immunomodulation (11–13). In the previous study, we have isolated a marine *Rhodotorula* sp., which can produce carotenoids (14), in this study, a novel extracellular acidic polysaccharide (EAPS) from marine *Rhodotorula* sp. RY1801 was purified, and its biological activities, such as antioxidant, nematode life extension, and immunomodulation, were investigated. The findings of the research suggest that EAPS from marine *Rhodotorula* sp. RY1801 would be an excellent microbial source of prebiotics, antioxidants, and immunomodulators for health promotion. The EAPS exhibited great potential to be developed into a valuable additive for the food and pharmaceutical industries.

## Materials and Methods

### Yeast, Bacterial Strains, *Caenorhabditis Elegans*, and Culture Conditions

The marine *Rhodotorula* RY1801 (CGMCC 15980) used in this study was isolated from the South Yellow Sea, Yancheng, China by the laboratory in the previous study (14). Bacteria used in this study include *Lactobacillus acidophilus* CGMCC1.2686, *Lactobacillus plantarum* ATCC8014, *Escherichia coli* OP50, and *Klebsiella pneumoniae* ATCC70063. *Rhodotorula* RY1801 were grown in Martin Broth at 30°C. *E. coli* and *K. pneumoniae* were grown in Luria-Bertani (LB) broth at 37°C with 120 rpm for 18–24 h. *L. acidophilus* and *L. plantarum* were grown in de Man, Rogosa, and Sharpe Broth (MRS Broth) at 37°C under anaerobic conditions. For long-term storage, bacteria were preserved at −70°C in LB containing 20% (v/v) glycerol.

For crude polysaccharides extraction, RY1801 was inoculated in the broth medium containing 5% glucose, 0.1% (NH_4_)_2_SO_4_, 0.1% KH_2_PO_4_, CaCl_2_ 0.01%, 0.04% NaCl, and 0.1% MgSO_4_∙7H_2_O at 10% inoculum concentration and incubated at 28°C in a rotary shaker at 200 rpm for 5 days.

*Caenorhabditis elegans* N2 (Bristol) was propagated under standard conditions, synchronized by hypochlorite bleaching, and cultured on nematode growth medium (NGM) at 16–20°C using *E. coli* strain OP50 as a standard food source.

### Materials and Chemicals

The Martin Broth, MRS, and NB mediums were purchased from Aoboxing Bio-Tech (Beijing, China). The Trizol Reagent was obtained from Thermo Fisher Scientific (Waltham, MA, USA). The One-Step TB Green PrimeScript™ RT-PCR Kit (Perfect Real-Time) was bought from TaKaRa (Shiga, Japan). The pyrogallol was purchased from Sangon Biotech (Shanghai, China) and the sodium salicylate from Sinopharm Chemical Reagent (Shanghai, China). Diethylaminoethyl (DEAE)-cellulose 52 and Sephadex G-200 were purchased from Fujian Phygene Biological Technology Co., Ltd. (Fujian, China).

### Extraction, Purification, and Characteristics of EAPS

Crude polysaccharides were extracted *via* previously reported methods (11). The fermentation broth was collected by centrifugation at 10,000 × g for 10 min. The cell-free supernatants were mixed with four volumes of 95% ethanol, stirred vigorously, and kept overnight at 4°C, and then centrifuged (10,000 × g, 10 min). The precipitate was dissolved in distilled water, and deproteinated by the Sevag method (chloroform: N-butanol at a 4:1) (15), followed by concentration, dialysis (cut-off 3,500 Da), and lyophilization. The crude EPS were obtained. The crude EPS was then purified using an anion exchange chromatography column (1.6 × 30 cm) with DEAE-cellulose 52 (11). The neutral fraction was first eluted with distilled water at 1 ml/min and then the acidic fractions were eluted with linear NaCl gradients (0–0.4 M) at 1 ml/min. The eluates were collected by an automatic collector (2 ml/tube). Collected fractions eluted with distilled water were obtained as extracellular neutral polysaccharides (ENPS). The fractions eluted with NaCl solution were obtained as an EAPS. The EAPS was then purified using a size exclusion chromatography column (1.5 × 60 cm) with Sephadex-G200. Gradient elution was conducted with distilled water at a flow rate of 0.3 ml/min, and the eluates were collected 1.5 ml/tube by an automatic collector. Collected fractions eluted with distilled water were concentrated and freeze-dried to obtain higher purity polysaccharides. The phenol-sulfuric acid method was used to determine the total sugar content of the eluent (16).

The molecular weights of EAPS were determined on high-performance gel permeation chromatography (HPGPC) *via* an Agilent-1200 high-performance liquid chromatographic (HPLC) system matched with a TSK gel G4000 PWxl column (7.8 × 300 mm), column temperature 30°C, and detected using a differential refraction index detector (RID) at 35°C as described by Zhang et al. (17).

The monosaccharide compositions were analyzed by HPLC after precolumn-derivatization of the hydrolysate with 1-phenyl-3-methyl-5-pyrazolone (PMP) according to the method of Honda et al. (18). Nine monosaccharides: mannose, ribose, rhamnose, glucuronide, glucose, galactose, xylose, arabinose, and fucose were selected as standard monosaccharides. Data were analyzed by the Chromeleon software.

### Prebiotic Effect of EAPS

We used acid polysaccharide EAPS to investigate its growth-promoting effects on *L. acidophilus* and *L. plantarum* (19). Basic MRS containing different concentrations of fructooligosaccharides (FOS) (Hefei Bomei Biotechnology Co., Hefei, China) and EAPS were used to test the prebiotic activity. About 1 × 10^6^ CFU/ml bacterial culture was added to the medium and incubated at 37°C for 48 h. The bacterial count was determined using the plate counting method, three independent experiments were performed, and the bacteria count was rescored as log_10_ CFU/ml.

### Assay of Antioxidant Activities *in vitro* of EAPS

#### Hydroxyl Radical Scavenging Activity

The hydroxyl radical scavenging activities of EAPS were determined according to the method by Wang et al. (20) with slight modification. About 1 ml of polysaccharide solution was mixed with 0.5 ml 1.5 mM FeSO_4_, 0.35 ml 6 mM H_2_O_2_, 0.15 ml 20 mM sodium salicylate, and then incubated at 37°C for 1 h. Then, the A_510_ value was detected, and the hydroxyl radical scavenging ability was calculated following the formula:

$$\text{Hydroxyl radical scavenging effect }(\%)=\left(1-\frac{A_{1}}{A_{0}}\right)\;\times 100\%$$

where A_1_ is the absorbance of the hydroxylated salicylate complex with the test sample, and A_0_ is with replacement distilled water. Ascorbic acid was used as a positive control in the same concentrations as that of the sample.

#### Superoxide Anion Radical Scavenging Activity

We used the pyrogallol autoxidation method to determine the superoxide anion radical scavenging ability of EAPS (21). Briefly, took 2.95 ml of Tris–HCl buffer [0.05 mM, pH 7.4, with 1 mM Na_2_ ethylenediaminetetraacetic acid (EDTA)] into a quartz cuvette, added about 0.05 ml of the pyrogallol solution (60 mM, soluble in 1 mM HCl), mixed quickly, started timing, and read the A value (325 nm) at 30 and 300 s (blank reference: Tris–HCl buffer). ΔA_0_ = A300s–A30s, where A300s and A30s are the absorbance at 300 and 30 s, respectively. Took 0.05 ml of each polysaccharide solution into a large quartz cuvette, added 2.9 ml Tris–HCl buffer, 50 μl of pyrogallol solution, mixed quickly. As above, the A_325_ value was measured in the 30 and 300 s, respectively (blank reference: Tris–HCl buffer). ΔA_1_ = A300s–A30s. Finally, used the followed formula to calculate the scavenging rate:

Scavenging ratio of superoxide anion: $(\frac{\Delta A_{0}-\Delta A_{1}}{\Delta A_{0}}\times \;100\%$

Ascorbic acid was used as a positive control in the same concentrations as that of the sample.

### Determination of Mean Lifespan of *C. elegans*

The influence of EAPS on the longevity of *C. elegans* was determined as described previously by Yaguchi et al. (22). Sixty synchronized nematodes were transferred to NGM plates spread with *E. coli* OP50 containing different concentrations of EAPS and 5-fluorodeoxyuridine and cultured at 20°C. The number of nematode survivors was counted daily, and the survival rate was calculated until all nematodes died.

#### Resistance Against *K. pneumoniae* Infection

The protective effect of EAPS on *K. pneumoniae* infection was tested (23). Plates with different concentrations of 0.6 mg/ml EAPS and 5-fluorodeoxyuridine were prepared by spreading 100 μl of overnight cultured *K. pneumoniae* ATCC70063. The plates were incubated at 37°C for 18–24 h to grow the bacterial lawn. *E. coli* OP50 plates were used as the negative control. Twenty synchronized L4 nematodes were transferred to each plate and incubated at 20°C, the number of worms was recorded for 96 h by visual observation of plates under a microscope. Each group had three replicates.

#### RNA Isolation and Quantitative Real-Time PCR

Nematodes fed with *E. coli* OP50 or *K. pneumoniae* were harvested and washed with sterile M9 solution two times, and total mRNA was isolated using the TRIzol Reagent (Thermo Fisher Scientific, Waltham, MA, USA). The primer sequences used in this study are shown in Table 1 (24). The quantitative real-time PCR (qRT-PCR) was performed using the Applied Biosystems 7500 Fast Real-Time PCR System (Thermo Fisher Scientific, Waltham, MA, USA) with the One-Step TB Green PrimeScript™ RT-PCR Kit (TaKaRa Shiga, Japan), the reverse transcription portion was 42°C, 5 min, 95°C, 10 s, and then the PCRs were carried out following conditions: 95°C at 5 s, 60°C at 34 s and cycled 40 times. Relative expression levels were calculated using the 2^−Δ*ΔCT*^ threshold cycle method. The control gene *act-2* was used to normalize the expression data of genes.

**Table 1:** The primer sequence used in this study.

**Gene**	**Primer**	**Sequence**
*act-2*	Forward	5′-CCCACTCAATCCAAAGGCTA-3′
	Reverse	5′-GGGACTGTGTGGGTAACACC-3′
*daf-2*	Forward	5′-GCCCGAATGTTGTGAAAACT-3′
	Reverse	5′-CCAGTGCTTCTGAATCGTCA-3′
*daf-16*	Forward	5′-TCCTCATTCACTCCCGATTC-3′
	Reverse	5′-CCGGTGTATTCATGAACGTG-3′
*pmk-1*	Forward	5′-CGAAGTGTCTGGGTTGTTCC-3′
	Reverse	5′-CGATATGTACGACGGGCATG-3′
*dbl-1*	Forward	5′-ATTGGCTGCTCGAAATGCTC-3′
	Reverse	5′-GGCGGTCACATCAAAATCGA-3′
*sma-3*	Forward	5′-CCACCGCACAGTCAATTATG-3′
	Reverse	5′-ATCTTCTCCCCAGCCCTTTA-3′

### Statistical Analysis

The experiments were repeated three times. Data analysis and mapping using the Graphpad 8.3 software. Statistical differences between groups were determined using one-way ANOVA. The level of *p* < 0.05 was considered to be statistically significant, and the level of *p* < 0.01 was considered to be statistically extremely significant.

## Results and Discussion

### Isolation, Purification, and Characterization of EAPS

**Figure 1:**
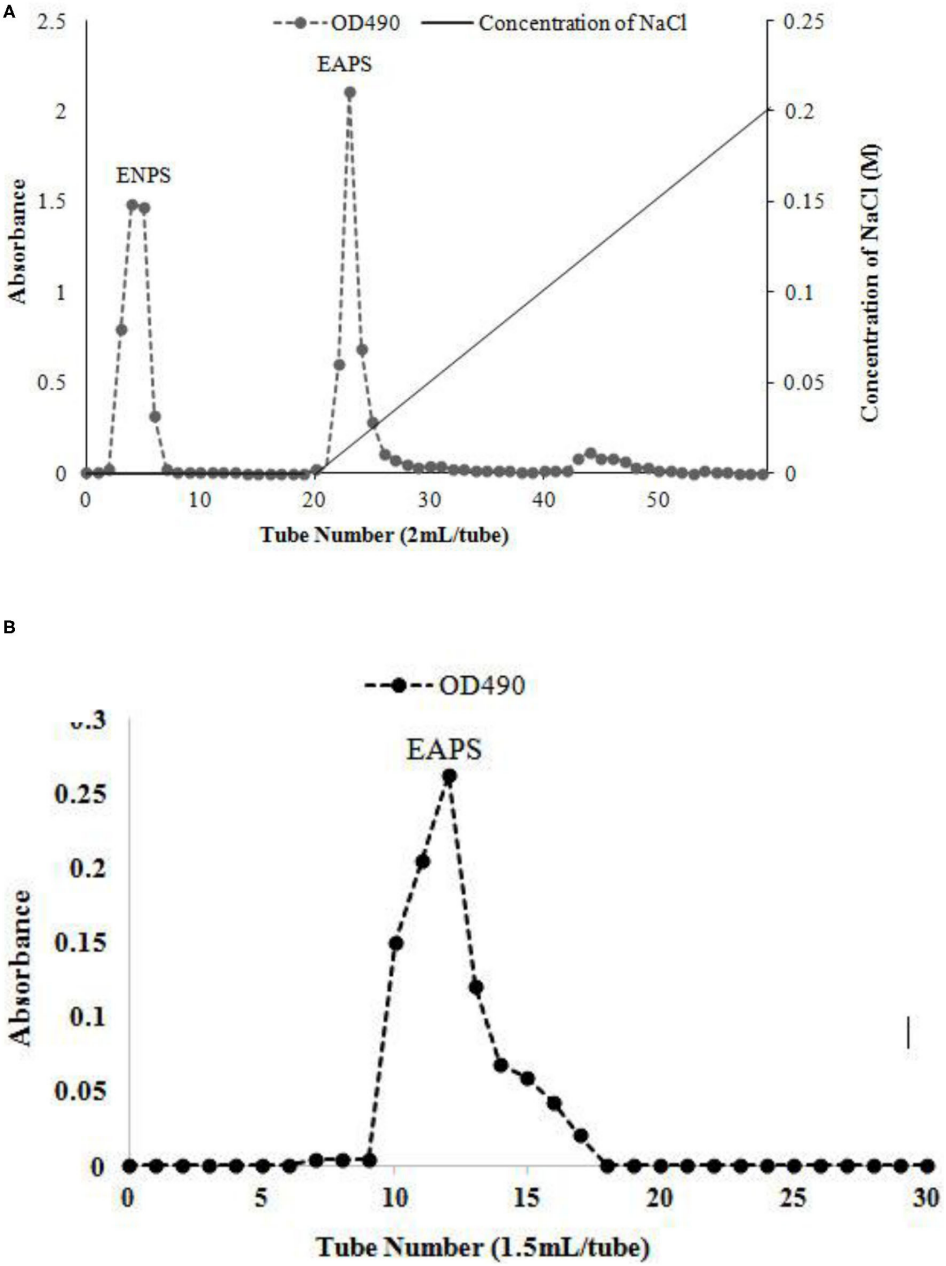
Elution curve of the water-extractable polysaccharide on the diethylaminoethyl (DEAE)-cellulose 52 anion exchange chromatography **(A)** and size exclusion chromatography **(B)**.

The extracellular polysaccharide was obtained from marine *Rhodotorula* RY1801 by alcohol precipitation, dialysis, and lyophilization. DEAE-cellulose 52 anion exchange chromatography was performed, and one neutral polysaccharide fraction and one acidic polysaccharide fraction were obtained, the EPS in each concentration was shown in Figure 1A. Fractions 3–8 were defined as ENPS, fractions 22-30 as EAPS. We purified EAPS using a Sephadex G200 gel column, and the fractions (10–19 tubes) were collected (Figure 1B), dialyzed, and lyophilized to obtain the purified EAPS for the following analysis.

**Figure 2:**
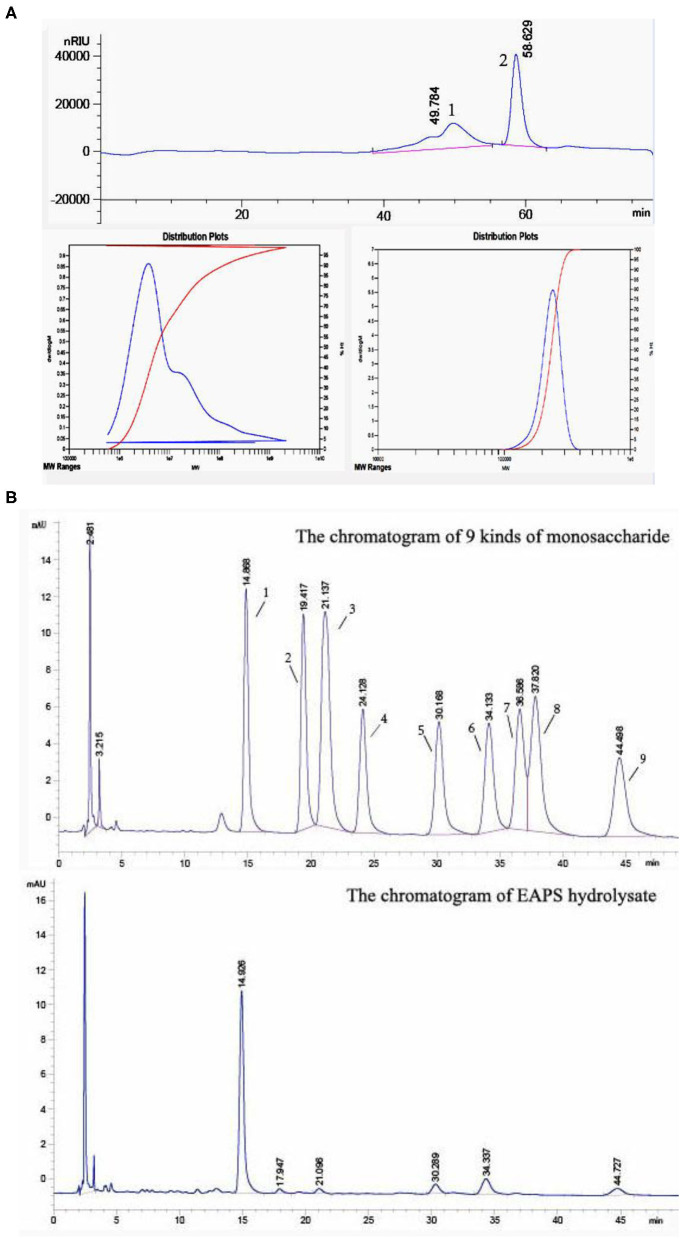
Homogeneity, molecular mass, and monosaccharide composition determination of extracellular acidic polysaccharide (EAPS). **(A)** High-performance gel permeation chromatography of the EAPS. **(B)** The monosaccharide composition of EAPS from marine *Rhodotorula* sp. RY1801.

The molecular weight of the EAPS was determined by using HPGPC. As shown in Figure 2A, two relatively homogeneous fractions were obtained at the retention time of 49.784 and 58.629 min. The molecular of the dominant component (peak 2) was estimated to be 5.902 × 10^7^ Da. In contrast, the molecular of the small peak was estimated to be 2.340 × 10^5^ Da. The biological activities of polysaccharides depending on the molecular weight and monosaccharide composition. Previously found that polysaccharides with a molecular weight of about 50–200 kDa have biological activities (25).

The monosaccharide composition of EAPS was analyzed by pre-column derivatization HPLC. It was found that EAPS is mainly composed of mannose, rhamnose, glucose, galactose, and fucose compared with standard spectra (Figure 2B). The molar ratio mannose:rhamnose:glucose:galactose:fucose is 44.9:1.0:3.7:8.0:4.6. Some researchers found that glucose and mannose are the major bioactive components in polysaccharides, which play essential roles in biological activities (26, 27). In this study, we found mannose is the main monosaccharide in EPAS. Therefore, the EPAS from marine *Rhodotorula* RY1801probably expresses some biological activities because of the high composition of mannose.

Several *Rhodotorula* spp. are capable of producing EPS with different monosaccharide compositions but mainly consist of mannose (27). *Rhodotorula minuta* has been confirmed as a safe source of EPS (28). Exopolysaccharide from *Rhodotorula mucilaginosa* CICC 33013 was composed of galactose, arabinose, glucose, and mannose (11). EPS of *R. minuta* ATCC 10658 consisted of mannose and glucose at a molar ratio of about 1.4:7.7 (29). The compositions of exopolysaccharides from a new cold-adapted *R. mucilaginosa* sp. GUMS16 are glucose and mannose at molar ratio of 5.7:1 (30). The monosaccharide composition of the exopolysaccharide produced by *Rhodotorula acheniorum* MC was 92.8% mannose and 7.2% glucose (31). In this study, we found the monosaccharide compositions of marine red yeast *Rhodotorula* RY1801 EPSs are mainly mannose, rhamnose, glucose, galactose, and fucose.

### Prebiotic Effects of EAPS

**Figure 3:**
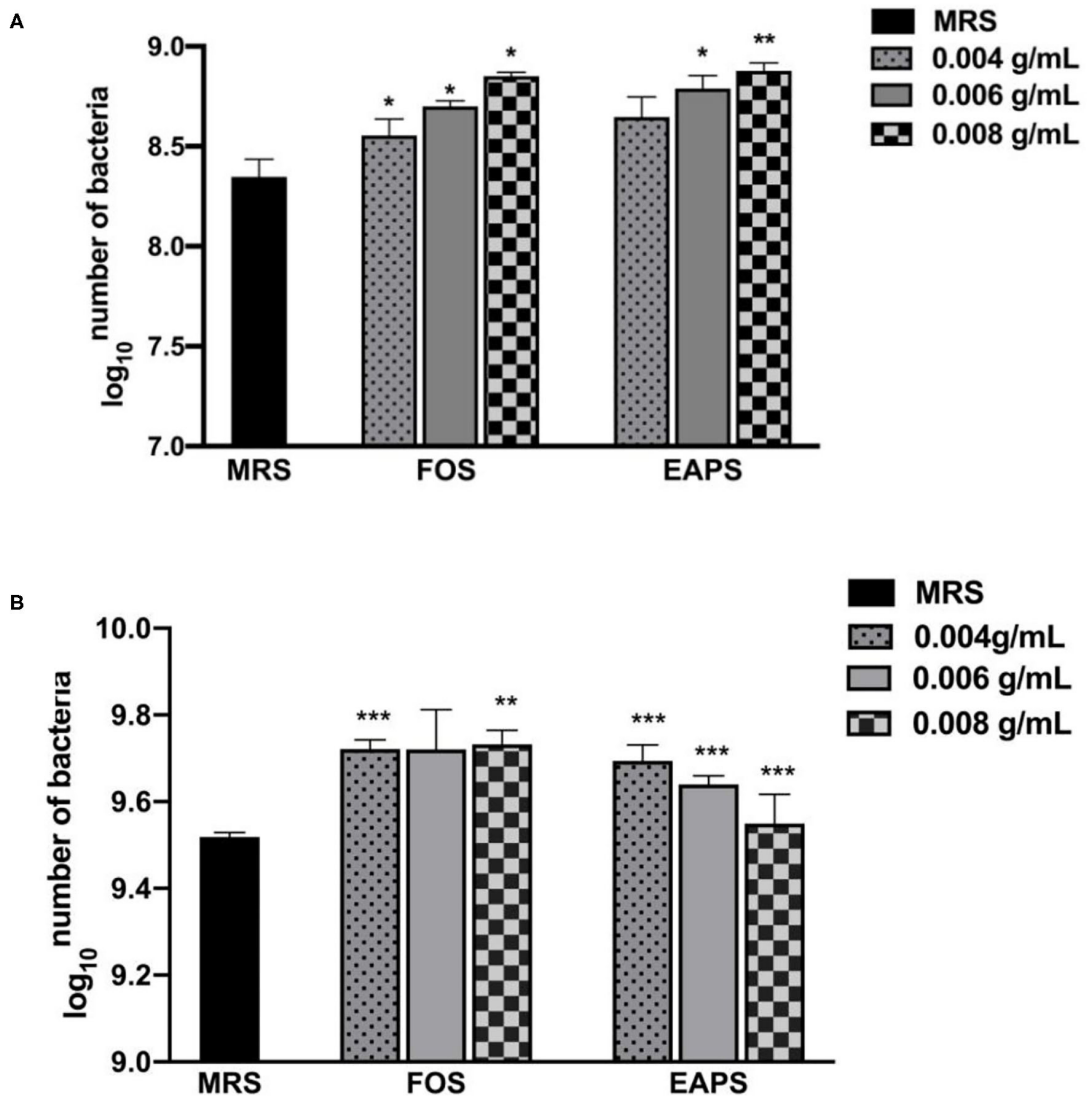
Prebiotic effects of EAPS on *Lactobacillus acidophilus* **(A)** and *Lactobacillus plantarum* **(B)**. de Man, Rogosa, and Sharpe Broth (MRS Broth) is the blank control; fructooligosaccharides (FOS) are a positive control. All tests were performed in triplicate and repeated at least once, and the results were expressed by their means ± SD. Statistical significance of the treatment effects was determined by Duncan's multiple range *t*-test (*p < 0.05, **p < 0.01, and ***p < 0.001).

We used EAPS as supplemental carbon sources to investigate its growth promotion effects on two probiotic stains, *L. acidophilus* and *L. plantarum*. EAPS showed prebiotic activity, which can promote the growth of *L. acidophilus* and *L. plantarum* (Figures 3A,B). FOS as the positive control also showed a significant promotion of *L. acidophilus* and *L. plantarum* growth. Various polysaccharides have been reported to show prebiotic activity, such as rapeseed polysaccharides can promote the growth of *Bifidobacterium bifidum, Bifidobacterium adolescentis, L. acidophilus*, and *Bifidobacterium infantis* (32). Soluble polysaccharides of palm kernel cake were found to support the growth performance of *L. plantarum* ATCC 8014 and *L. rhamnosus* ATCC 53103 (33). A long linear chain of dextran produced by *Weissella cibaria* enhanced the relative amount of *Prevotella* and *Bacteroides* (34). Polysaccharides isolated from *Grateloupia filicina* and *Eucheuma spinosum* can significantly promote the growth of *Bifidobacterium* (35). *Lycium barbarum* polysaccharide can support the growth of *L. acidophilus* and *B. longum*. After feeding mice with *L. barbarum* polysaccharide, sequencing of the intestinal flora of mice revealed that the abundance of the phyla Proteobacteria and Firmicutes was increased, which reduced the abundance of the phylum Bacteroidetes (36). In this study, the results demonstrated that EAPS promotes the growth of *L. acidophilus* and *L. plantarum* in a dose-dependent manner. These results could be explained by those polysaccharides can be used as a carbon and energy source by the microbial population.

### Assay of Antioxidant Activities *in vitro* of EAPS

**Figure 4:**
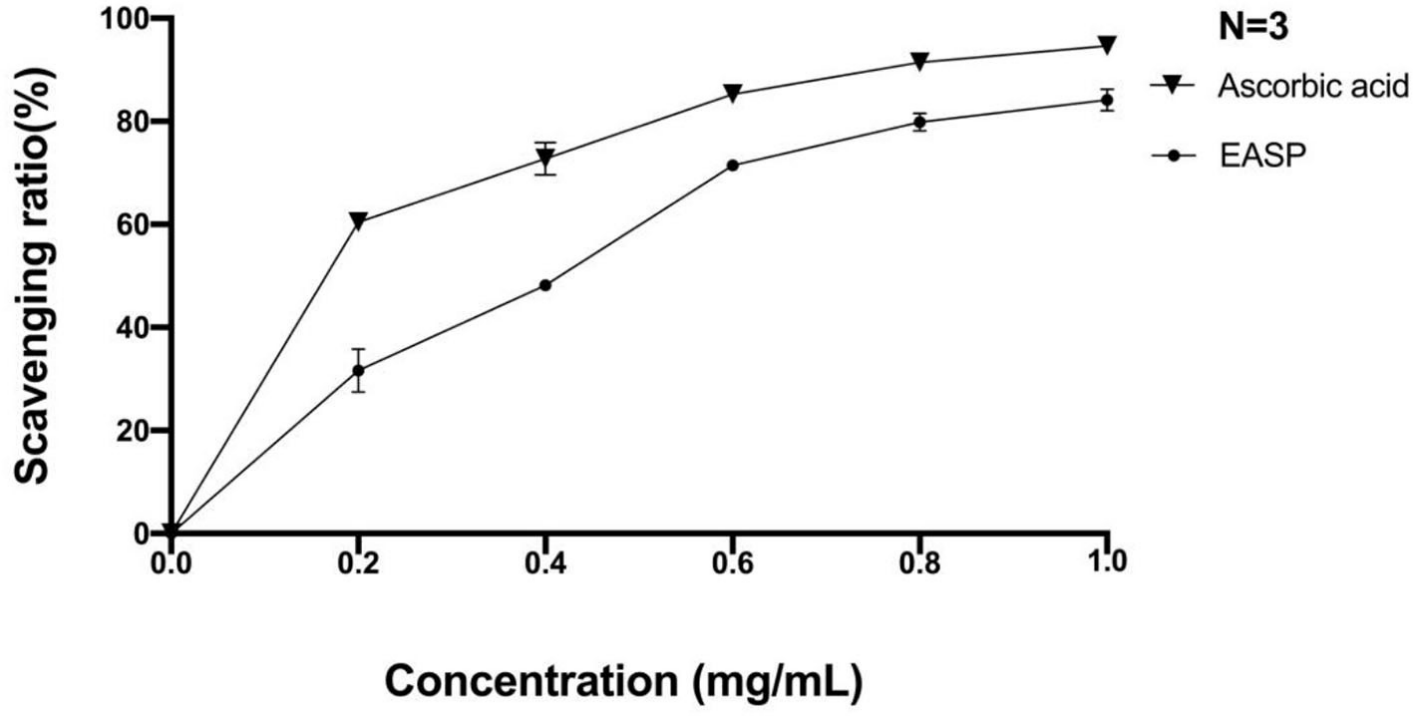
Scavenging effects of EAPS on hydroxyl free radical.

Hydroxyl radical is known to be the most harmful of active oxygens to living organisms. It can cause oxidative damage to amino acids, proteins, nucleic acids, and lipids (37). We used the salicylic acid method to determine the scavenging ability of EAPS to hydroxyl radicals. As shown in Figure 4, at 0.2 and 0.4 mg/ml, the scavenging rates of EAPS were 32 and 48%, respectively. As the concentration increased, so did the scavenging rate. The hydroxyl free radical scavenging of EAPS reached a maximum of 84% at a concentration of 1 mg/ml. A similar trend in the percent inhibition was also produced by ascorbic acid as the positive control.

**Figure 5:**
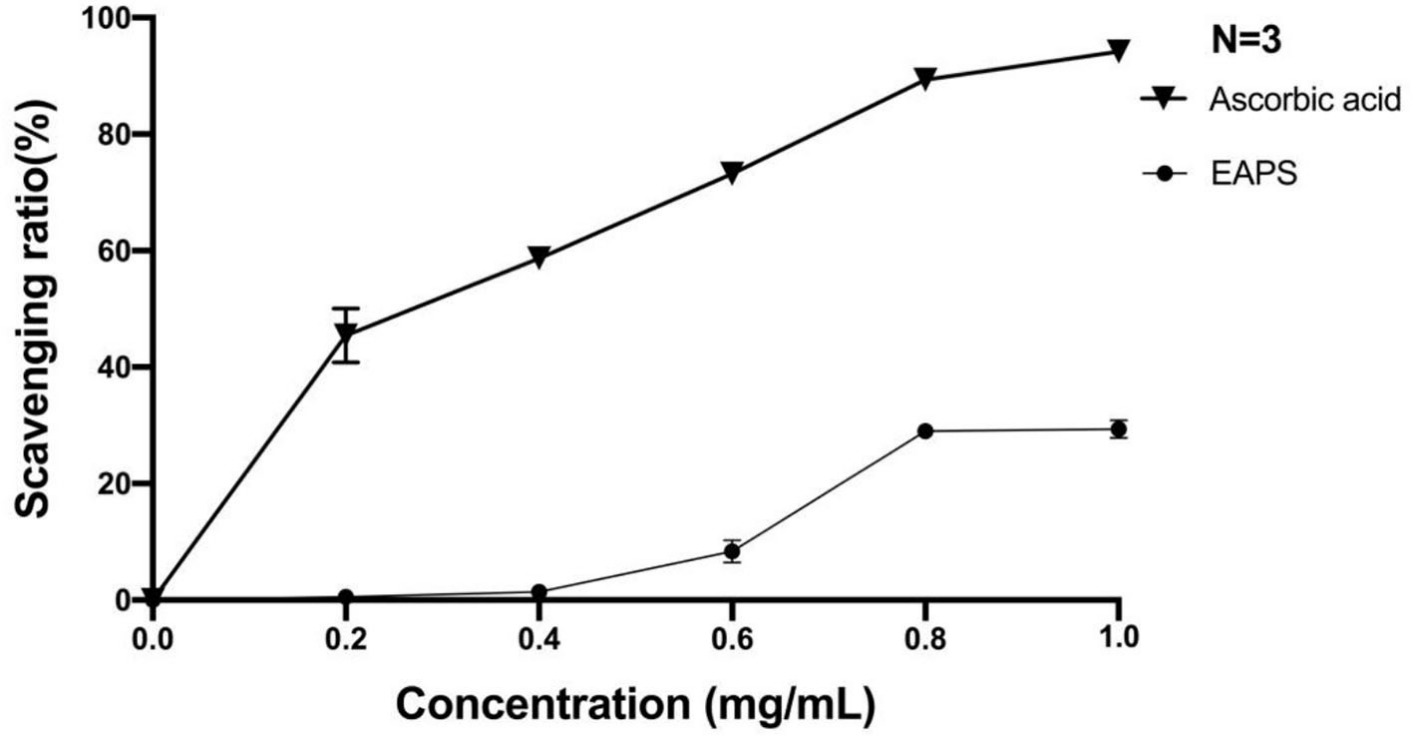
Scavenging effects of EAPS on superoxide anion radical.

Superoxide anion radical is one of the active oxygen free radicals generated in the human body, which plays an important role in several human diseases, such as lipid peroxidation, carcinogenesis, cancer, etc. In this study, we used the pyrogallol autoxidation method to determine the ability of EAPS to scavenge the superoxide anion radical. As shown in Figure 5, scavenging rates of superoxide anion radical by EAPS were low at concentrations of 0.2 mg/ml (0.5%) and 0.4 mg/ml (1.4%). At the concentration of 0.8 mg/ml, the clearance reached a maximum of 29%. Likewise, ascorbic acid, which was used as a positive control, also shown a concentration-dependent superoxide anion radical activity, and its activity is higher than EAPS.

Usually, the DPPH, OH, and O_2_-scavenging ratios were used to indicate the antioxidant capacity of polysaccharides *in vitro*. Various polysaccharides from different sources have shown antioxidant capacity (38). In this study, we found that EAPS derived from *Rhodotorula* RY1801 showed certain antioxidant capacity in a dose-dependent manner. Polysaccharides from *Epimedium acuminatum Franch* showed excellent ability to scavenge hydroxyl radicals and superoxide radicals, and it may be related to reactive hydroxyl groups in polysaccharides and uronic acid content, respectively (39). Extracellular and intracellular polysaccharides from *Fomitopsis pinicola* showed antioxidant activities in a dose-dependent manner (40). *In vitro*, marine red yeast polysaccharides obtained from *Rhodosporidium paludigenum* showed weak antioxidant activity compared with Vitamin C (41). Exopolysaccharides produced by *L. sanfranciscensis* Ls-1001 had high antioxidant activity and had a significant effect on the removal of ABTS free radicals (42). The antioxidant capacity of different polysaccharides mainly depends on their physicochemical features, including the molecular weight and monosaccharide composition (43).

### Effects of EAPS on the Lifespan of *C. elegans*

**Figure 6:**
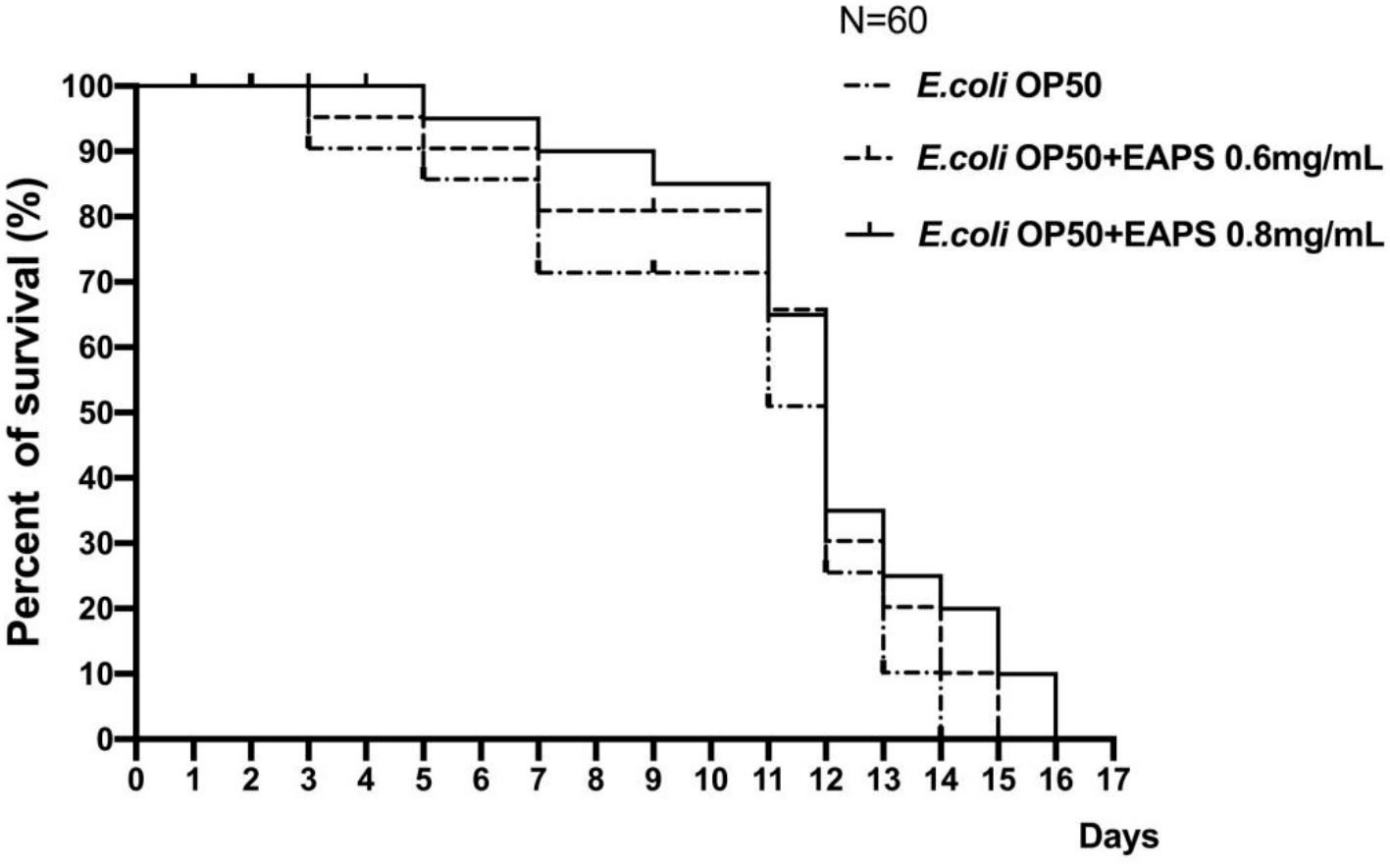
Survival curves of *Caenorhabditis elegans* after treatment with EAPS.

In this study, we found that the mean lifespan of nematodes in the EAPS 0.6 and 0.8 mg/ml groups was 11.23 ± 0.22 and 11.95 ± 0.20 days, respectively, an increase of 11.19 and 18.32% compared to the control group. As shown in Figure 6, no nematodes died in all groups in the first 2 days. On day 3, two nematodes died in the control group, only one nematode died in the EAPS 0.6 mg/ml group, and no nematode died in the EAPS 0.8 mg/ml group. In the EAPS 0.8 mg/ml group, only till day 5, the nematode began to die. Compared to the other groups, the initial death of EAPS 0.08 mg/ml was delayed by 2 days. On day 11, the survival rate of the control group was only 50%, whereas the other groups were >60%. Over time, all nematodes in the control group died on day 14. All nematodes in the EAPS 0.6 mg/ml group died on day 15. Finally, the group EAPS 0.8 mg/ml had no living nematodes on day 16. Overall, the nematodes of the EAPS 0.8 mg/ml group lived the longest.

There have been many previous studies on the effects of various foods on the life of nematodes. These foods include some prebiotics and even water extracts of natural plants (44, 45). However, it is rare to see polysaccharides used in life tests of nematodes, especially those derived from microorganisms. In this experiment, the EPSs were derived from the *Rhodotorula* RY1801 that was independently isolated and identified by the laboratory. When applied to nematodes, it was found that EAPS showed a certain life extension effect. EAPS showed a certain antioxidant capacity *in vitro*. In this experiment, the survival rate of nematodes was also improved to some extent, which seems to coincide. The life extension effect of EAPS maybe because of the antioxidant activity and prebiotic activity on gut microbiota which can promote the healthy function of the host intestinal system. The same results were found in other polysaccharides. The lifespan extension effect of polysaccharides from *Rehmannia glutinosa* may be related to its antioxidant activity (46). Polysaccharides (HWPs) from *Chlorella vulgaris* significantly increased the lifespan of *C. elegans* under oxidative stress induced by hydrogen peroxide (47). Some other studies showed that polysaccharides can modulate gut microbiota and then promote the longevity of *C. elegans* (48, 49).

### EAPS Attenuate the Pathogenicity of *K. pneumoniae* in *C. elegans*

**Figure 7:**
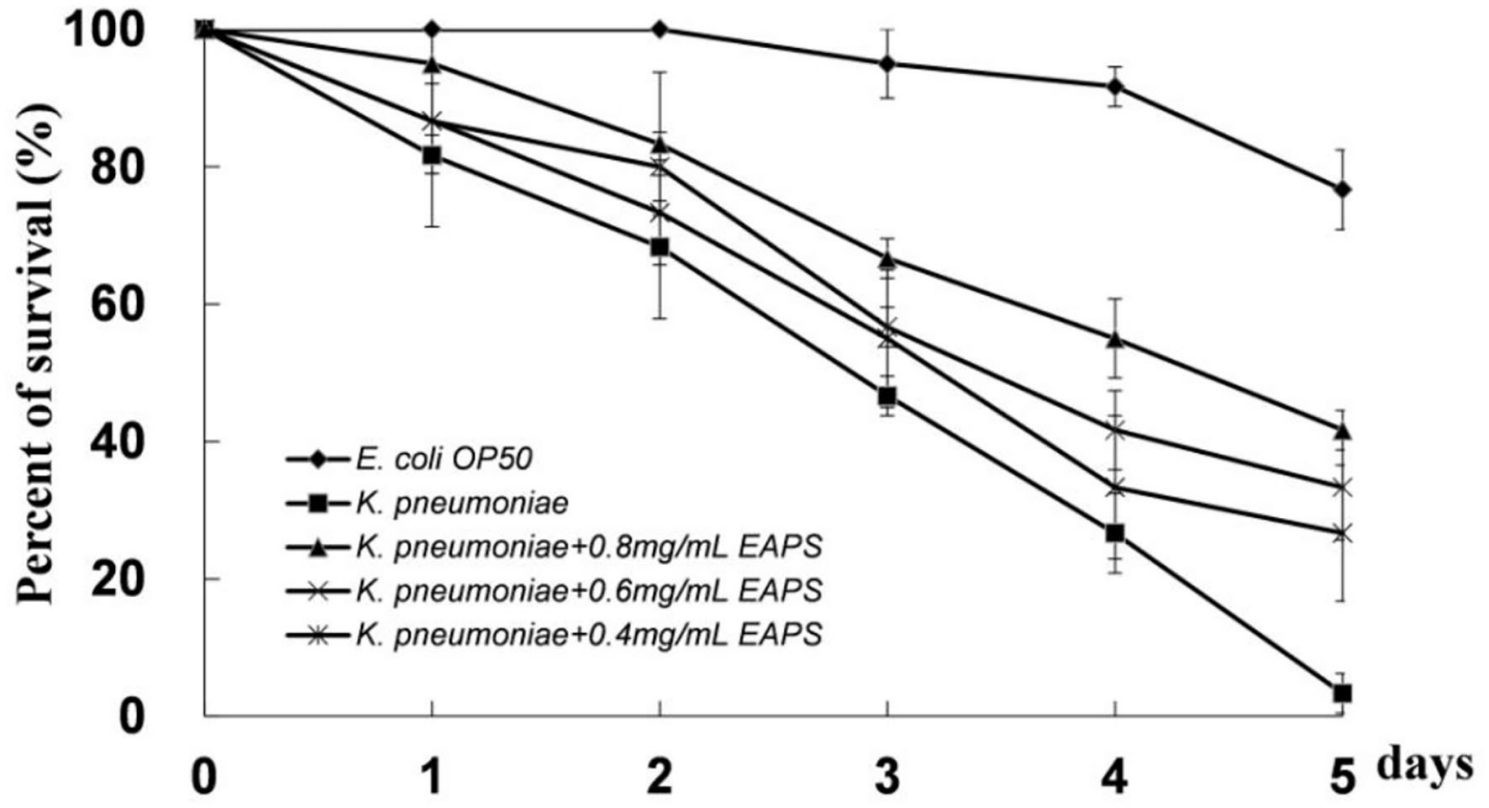
Protective effect of EAPS on *C. elegans* against *Klebsiella pneumoniae* infection. The graph represents the average of three independent experiments and an SD.

*Caenorhabditis elegans* provides an excellent model for studying host–bacteria interactions. We detected the protective effect of EAPS on *C. elegans* against *K. pneumoniae* infection. EAPS increased the survival of the infected *C. elegans* in a concentration-dependent manner (Figure 7). Polysaccharides can be used by beneficial bacteria, such as *Bifidobacterium* and *Lactobacillus*, and inhibit the growth of pathogenic bacteria. Microbial exopolysaccharides have been shown to stimulate the immune system and increase the disease-resistant of the host (50). A recent study suggests that polysaccharides from *Sophora moorcroftiana* seeds significantly extend the life span, improved reproduction, and increased the stress resistance and antimicrobial capacity of *C. elegans* (51).

### Relative Gene Expression in *C. elegans*

**Figure 8:**
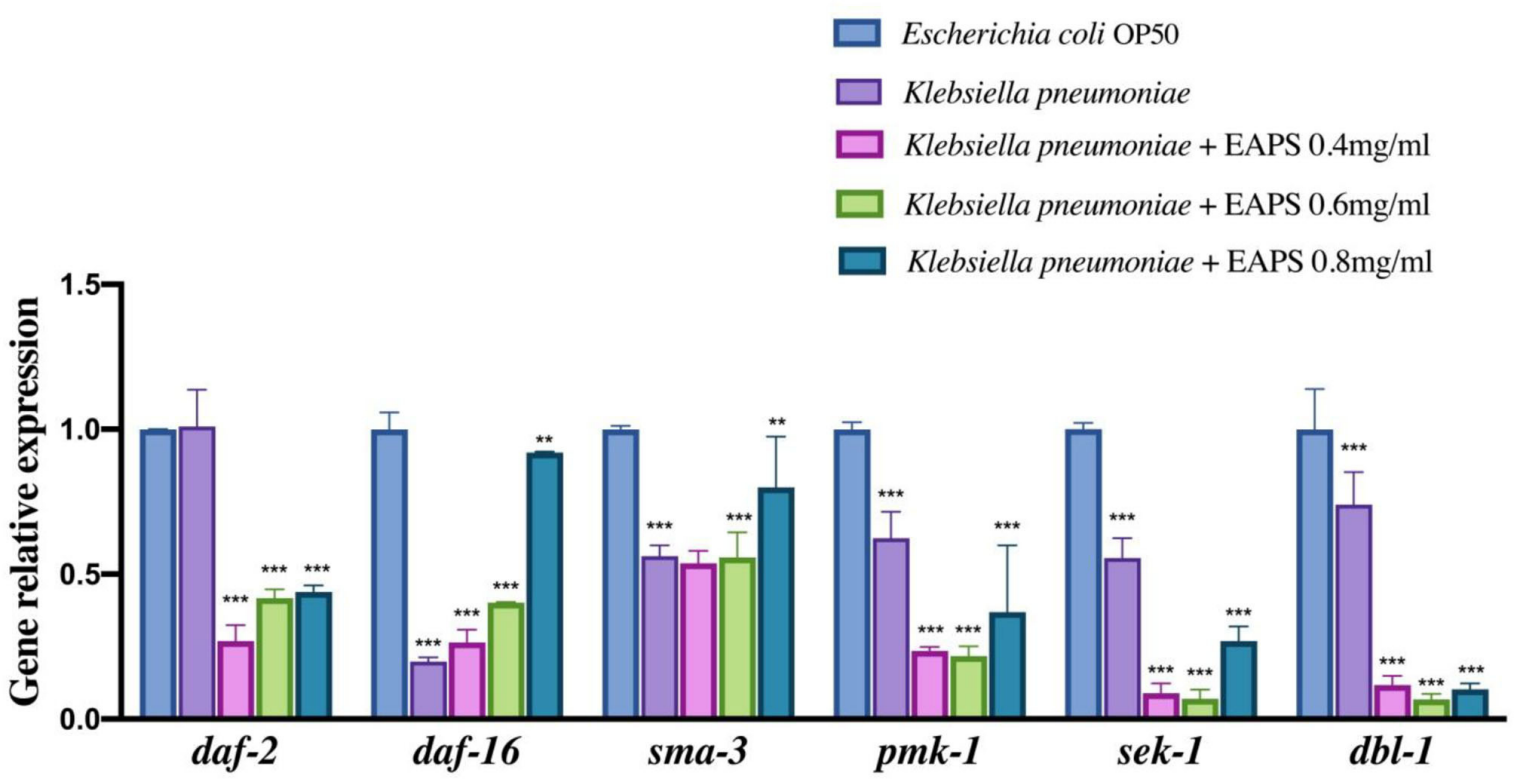
Relative expression of immunity and longevity-related genes in infected nematodes treated with EAPS. All tests were performed in triplicate and repeated at least once, and the results were expressed by their means ± SD. Statistical significance of the treatment effects was determined by Duncan's multiple range *t*-test (**p < 0.01 and ***p < 0.001).

The DAF-2/DAF-16 pathway is related to the lifespan regulation of nematodes (52). In this study, we examined the following genes related to nematode immunity and longevity: *daf-2, daf-16, dbl-1 pmk-1, sek-1*, and *sma-3* genes were used to investigate the effect of the EAPS on nematodes. The qPCR results are shown in Figure 8, the expression of *daf-16, dbl-1 pmk-1, sek-1*, and *sma-3* genes in the nematodes was significantly downregulated after *K. pneumoniae* infection. The relevant genes were re-examined after different concentrations of EAPS-treated infected nematodes, and *daf-2* gene expression was extremely significantly downregulated in the EAPS-treated infected nematode group compared to the infected nematode group and control. The daf-16 gene, which was significantly downregulated in infected nematodes, rebounded significantly after treatment with EAPS, particularly in the EAPS 0.8 mg/ml group. Similarly, the *sam-3* gene, which is downregulated in infected nematodes, was significantly regressed in the EAPS 0.8 mg/ml group. But the expression of the other three genes, *sek-1, pmk-1*, and *dbl-1*, was further suppressed after treatment with EAPS.

The nematode *C. elegans* is now a major model organism in biology research, such as lifespan, bacterial infection, and host innate immune. Although *C. elegans* may not much resemble humans, the molecules that regulate the immune response in these two organisms prove to be quite similar (53). Nematodes lack adaptive immunity and rely on innate immunity to respond to external threats, mainly including four conserved immune signaling pathways (54): (1) transforming growth factor (TGF)-β pathway; (2) the p38 mitogen-activated protein kinase (MAPK) pathway; (3) programmed cell death pathway; and (4) DAF-2/DAF-16 pathway. In the TGF-β pathway, dbl-1 is responsible for encoding the TGF-b receptor. The combination of the two causes phosphorylation of SMAD proteins (composed of *sma-2, sma-3*, and *sma-4*) and related antibacterial genes thereby activated (55). Sek-1 and mek-1 are related to the innate immunity of nematodes in the p38 MAPK pathway. They activate *pmk-1*, but the pathway has not been elucidated. The DAF-16 enhances the resistance to stress in *C. elegans*, which is important for aging. However, DAF-2 inhibits the production of DAF-16. In this experiment, infected nematodes were treated with EAPS and the *daf-16* gene was significantly regressed. Correspondingly, the *daf-2* gene was significantly downregulated. These results suggested that disease resistance of nematode is diminished after infection with *K. pneumoniae*. The nematode resistance was repaired to some extent after treatment with EAPS. It can be hypothesized that EAPS plays a role in the DAF-2/DAF-16 pathway of *C. elegans* and may increase DAF-16 production by inhibiting DAF-2 expression to increase nematode resistance and prolong life. Besides, treatment with EAPS upregulated the *sma-3* gene that was significantly downregulated after nematode infection and was not significantly different from the control group, which may indicate that the antibacterial capacity of *C. elegans* was restored. However, the previous literature reported differently whether *dbl-1*, an upstream gene that activates the *sma-3* gene in the TGF-β pathway, was further suppressed and whether EAPS is involved in other immune signaling pathways in nematodes and initiates expression of the *sma-3* gene. Downregulation of other genes *pmk-1* and *sek-1* may imply that EAPS does not act through the p38 MAPK pathway.

## Conclusions

In this study, we obtained water-extractable EAPS from marine *Rhodotorula* sp. RY1801 by DEAE-cellulose 52 anion exchange chromatography and Sephadex G200 chromatography. Results indicated that EAPS showed a growth promotion effect on the growth of *L. acidophilus* and *L. plantarum*. EAPS all possessed antioxidant activity in superoxide anion and hydroxyl radicals *in vitro*, respectively. EAPS could expand the lifespan and increase the disease resistance of *C. elegans* against *K. pneumoniae* infection. EAPS from marine *Rhodotorula* sp. RY1801 was found to be excellent microbial sources of prebiotics, antioxidants, and immunomodulators for health promotion. The EAPS exhibited great potential to be developed into a valuable additive for the food and pharmaceutical industries.

## Data Availability Statement

The original contributions presented in the study are included in the article/supplementary material, further inquiries can be directed to the corresponding author.

## Author Contributions

WC and ZW: conceptualization. YZ: methodology and data curation. ZW: software and writing the original draft preparation and review and editing. ZW and YZ: validation. ZW and YJ: formal analysis. YZ and YJ: investigation. WC: resources, visualization, supervision, project administration, and funding acquisition. All authors contributed to the article and approved the submitted version.

## Conflict of Interest

The authors declare that the research was conducted in the absence of any commercial or financial relationships that could be construed as a potential conflict of interest.

## Publisher's Note

All claims expressed in this article are solely those of the authors and do not necessarily represent those of their affiliated organizations, or those of the publisher, the editors and the reviewers. Any product that may be evaluated in this article, or claim that may be made by its manufacturer, is not guaranteed or endorsed by the publisher.
